# A second monoclinic polymorph of 2-[2-(4-meth­oxy­phen­yl)hydrazinyl­idene]-1,3-diphenyl­propane-1,3-dione

**DOI:** 10.1107/S1600536811024445

**Published:** 2011-06-30

**Authors:** Carlos Bustos, Luis Alvarez-Thon, Daniela Barría, Juan-Guillermo Cárcamo, Maria Teresa Garland

**Affiliations:** a Instituto de Ciencias Químicas, Universidad Austral de Chile, Avda. Los Robles s/n, Campus Isla Teja, Casilla 567, Valdivia, Chile; b Departamento de Ciencias Físicas, Universidad Andres Bello, Avda. República 220, Santiago de Chile, Chile; c Instituto de Ciencias Moleculares y Microbiología, Universidad Austral de Chile, Avda. Los Robles s/n, Campus Isla Teja, Casilla 567, Valdivia, Chile; d Laboratorio de Cristalografía, Departamento de Física, Facultad de Ciencias Físicas y Matemáticas, Universidad de Chile, Av. Blanco Encalada 2008, Santiago de Chile, Chile

## Abstract

The title compound, C_22_H_18_N_2_O_3_ is the second monoclinic polymorph (*P*2_1_/*c*) of the compound, the first being reported in space group *P*2_1_ [Bertolasi *et al.* (1993 ▶). *J. Chem. Soc. Perkin Trans. 2*, pp. 2223–2228]. In the mol­ecular structure of the title compound, the inter­planar angle between the benzoyl units is 80.04 (5)°, while the corresponding angles between the phenyl­hydrazinyl­idene and benzoyl groups are 36.11 (5) and 55.77 (2)°. A strong resonance-assisted intra­molecular N—H⋯O hydrogen bond is found. In the crystal, the entire supra­molecular structure is constructed by weak inter­molecular C—H⋯O inter­actions and an inter-ring π–π inter­action [centroid–centroid distance = 3.6088 (8) Å].

## Related literature

For details of the synthesis, see: Yao (1964 ▶). For resonance-assisted hydrogen bonds and related structures, see: Bertolasi *et al.* (1993 ▶); Bustos, Alvarez-Thon, Barría *et al.* (2011 ▶); Bustos, Alvarez-Thon, Cárcamo, Garland & Sánchez (2011 ▶).; Bustos, Alvarez-Thon, Cárcamo, Ibañez & Sánchez (2011 ▶); Gilli *et al.* (1993 ▶).
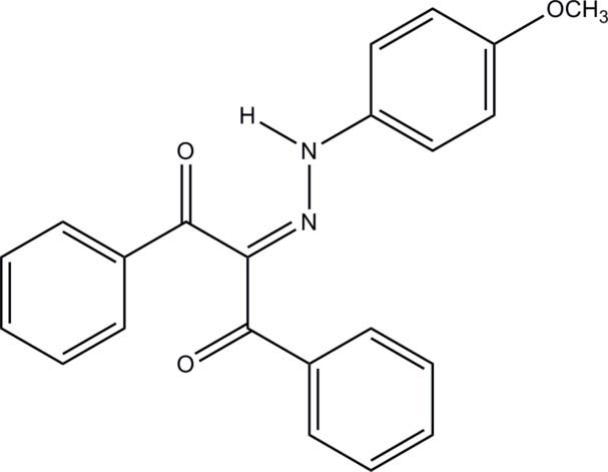


## Experimental

### 

#### Crystal data

C_22_H_18_N_2_O_3_
*M* *_r_* = 358.38
Monoclinic, 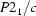

*a* = 12.3045 (11) Å
*b* = 11.055 (1) Å
*c* = 14.2435 (13) Å
β = 113.683 (1)°
*V* = 1774.3 (3) Å^3^
*Z* = 4
Mo *K*α radiation
μ = 0.09 mm^−1^
*T* = 150 K
0.52 × 0.26 × 0.15 mm

#### Data collection

Bruker D8 Discover diffractometer with SMART CCD area detector
10646 measured reflections
3600 independent reflections
2894 reflections with *I* > 2σ(*I*)
*R*_int_ = 0.036

#### Refinement

*R*[*F* ^2^ > 2σ(*F* ^2^)] = 0.039
*wR*(*F* ^2^) = 0.105
*S* = 1.04
3600 reflections
249 parameters
H atoms treated by a mixture of independent and constrained refinement
Δρ_max_ = 0.26 e Å^−3^
Δρ_min_ = −0.18 e Å^−3^

### 

Data collection: *SMART* (Bruker, 2001 ▶); cell refinement: *SAINT* (Bruker, 2000 ▶); data reduction: *SAINT*; program(s) used to solve structure: *SHELXS97* (Sheldrick, 2008 ▶); program(s) used to refine structure: *SHELXL97* (Sheldrick, 2008 ▶); molecular graphics: *XP* in *SHELXTL-PC* (Sheldrick, 2008 ▶); software used to prepare material for publication: *PLATON* (Spek, 2009 ▶) and *Mercury* (Macrae *et al.*, 2006 ▶).

## Supplementary Material

Crystal structure: contains datablock(s) global, I. DOI: 10.1107/S1600536811024445/zs2122sup1.cif

Structure factors: contains datablock(s) I. DOI: 10.1107/S1600536811024445/zs2122Isup2.hkl

Supplementary material file. DOI: 10.1107/S1600536811024445/zs2122Isup3.cml

Additional supplementary materials:  crystallographic information; 3D view; checkCIF report

## Figures and Tables

**Table 1:** Hydrogen-bond geometry (Å, °)

*D*—H⋯*A*	*D*—H	H⋯*A*	*D*⋯*A*	*D*—H⋯*A*
N2—H1⋯O2	0.884 (14)	1.977 (15)	2.6427 (14)	131.0 (14)
C15—H15⋯O3^i^	0.95	2.64	3.5859 (16)	174
C21—H21⋯O1^ii^	0.95	2.63	3.3441 (15)	132

Symmetry codes: (i) 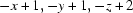; (ii) 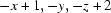.

**Fig. 1.**
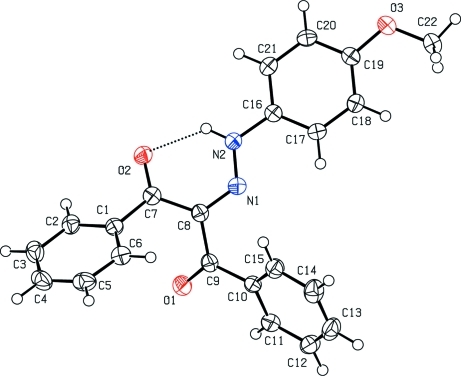
View of the title compound with the atom numbering scheme. Displacement ellipsoids for non-H atoms are drawn at the 50% probability level. The strong intramolecular hydrogen bond (N2–H1···O2) is depicted with a dashed line.

**Fig. 2.**
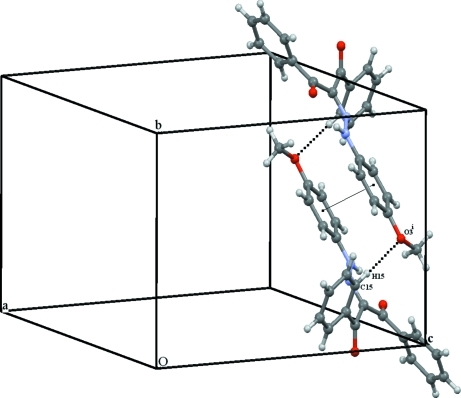
Part of the crystal packing showing the inversion-related interaction via C15—H15···O3i weak contacts (dashed lines) and a π-π stacking interaction Cg···Cgi (solid line) [symmetry code: (i) -x + 1, -y + 1, -z + 2].

**Fig. 3.**
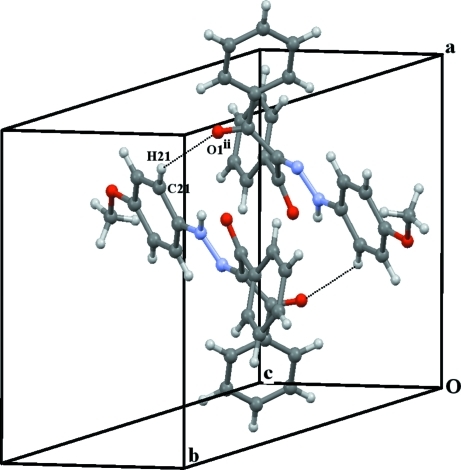
Part of the crystal packing showing the inversion-related interaction via C21—H21···O1ii contacts (dashed lines) [(ii) -x + 1, -y, -z + 2]].

## supplementary crystallographic information

### Comment

Extensive experimental and theoretical efforts have been devoted to understand the reasons for the formation of the very strong intramolecular O—H···O hydrogen bond in β-diketoenolic systems (Bertolasi *et al.*, 1993). A model called RAHB (Resonance Assisted Hydrogen Bond) was suggested, which is essentially a synergistic mutual reinforcement of the H-bond with the π-delocalization within this heterodienic system (Gilli *et al.*, 1993). Besides, this concept has been applied to other heterodienic systems such as enaminones and ketohydrazones. On the other hand, in previous works we have reported the crystal structures of three β-diketohydrazones of the type 2-(2-(*R*-phenyl)hydrazinylidene)-1,3-diphenylpropane-1,3-dione (*R* = 4-Br, 4-NO_2_, 3-Cl) that contain this type of hydrogen bond (Bustos, Alvarez-Thon, Barría *et al.*, 2011; Bustos, Alvarez-Thon, Cárcamo, Garland & Sánchez, 2011; Bustos, Alvarez-Thon, Cárcamo, Ibañez & Sánchez, 2011. We now present the crystal structure of a second monoclinic polymorph of the compound C_22_H_18_N_2_O_3_ (I), described previously in the literature (Bertolasi *et al.*, 1993), prepared using similar methodology (Yao, 1964) and recrystallizing from ethanol as orange crystals. The title compound crystallizes in space group *P*2_1_/*c* whereas the previously reported structure (Bertolasi *et al.*, 1993) crystallizes in space group *P*2_1_. The most striking structural difference between the two polymorphs is in the methoxy group which appears rotated by 180°.

In the molecular structure of the title compound (Fig. 1), the interplanar angle between the benzoyl units is 80.04 (5)°. The corresponding angles between the phenylhydrazinylidene and the benzoyl groups are 36.11 (5) and 55.77 (2)°, respectively. In the crystal, a strong resonance-assisted intramolecular hydrogen bond, N2—H1···O2, is observed (Table 1).

The structure shows no conventional intermolecular hydrogen bonds and the entire supramolecular structure is constructed only by weak interactions. For the sake of clarity, the crystal packing can be described through the formation of two inversion-related dimers: firstly, a dimer is formed *via* weak intermolecular contacts of the type C15—H15···O3^i^, and a π-π stacking interaction with a Cg···Cg^i^ distance of 3.6088 (8) Å, where Cg is the centroid of the C16—C21 ring (Fig. 2) [symmetry code: (i) -*x* + 1, -*y* + 1, -*z* + 2]. Secondly, a dimer is formed *via* weak contacts of the type C21—H21···O1^ii^ [3.3441 (15) Å] (Fig. 3) [symmetry code: (ii) -*x* + 1, -*y*, -*z* + 2].

### Experimental

Chemicals: 1,3-diphenylpropane-1,3-dione, *p*-anisidine and sodium nitrite were procured from Sigma-Aldrich and sodium hydroxide, hydrochloric acid, sodium acetate and solvents from Merck. These chemicals were used without previous purification.

Procedure: A solution of 2.29 g (0.01 mole) of 1,3-diphenylpropane-1,3-dione (98%) in 100 ml of ethanol solution containing 0.4 g (0.01 mole) of sodium hydroxide was buffered by adding 4.80 g of sodium acetate trihydrate. The resulting β-diketonate solution was diluted with water to a volume of about 220 ml and stirred and cooled to -5 °C. A diazonium ion solution was prepared adding 1.24 g (0.01 mole) of *p*-anisidine (99%) in 8 ml of hydrochloric acid (5 mol/*L*), cooled to -5 °C, and a saturated aqueous solution containing 0.69 g (0.01 mole) of sodium nitrite was added dropwise . This solution was then added dropwise with vigorous stirring into the buffered β-diketonate solution. During the addition a yellow precipitate was formed which was filtered by suction and washed with an abundant quantity of water. Yield: 95% of crude product. Orange single crystals suitable for X-ray studies were obtained by recrystallization from a concentrated solution of the compound in ethanol.

### Refinement

All hydrogen atoms were found in difference Fourier maps. The hydrogen attached to N2 was refined freely against the diffraction data, but all other H atoms were placed in geometrically idealized positions and constrained to ride on their parent atoms, with aromatic C—H = 0.95 Å, methyl C—H = 0.98 Å and *U*_iso_(H) = 1.2*U*_eq_ (aromatic C) or 1.5*U*_eq_(aliphatic C).

### Crystal data

**Table:**

C_22_H_18_N_2_O_3_	*F*(000) = 752
*M**_r_* = 358.38	*D*_x_ = 1.342 Mg m^−^^3^
Monoclinic, *P*2_1_/*c*	Mo *K*α radiation, λ = 0.71073 Å
Hall symbol: -P 2ybc	Cell parameters from 999 reflections
*a* = 12.3045 (11) Å	θ = 1.8–26.4°
*b* = 11.055 (1) Å	µ = 0.09 mm^−^^1^
*c* = 14.2435 (13) Å	*T* = 150 K
β = 113.683 (1)°	Polyhedron, orange
*V* = 1774.3 (3) Å^3^	0.52 × 0.26 × 0.15 mm
*Z* = 4	

### Data collection

**Table:**

Bruker D8 Discover diffractometer with SMART CCD area detector	2894 reflections with *I* > 2σ(*I*)
Radiation source: fine-focus sealed tube	*R*_int_ = 0.036
graphite	θ_max_ = 26.4°, θ_min_ = 1.8°
φ and ω scans	*h* = −14→15
10646 measured reflections	*k* = −13→13
3600 independent reflections	*l* = −17→17

### Refinement

**Table:**

Refinement on *F*^2^	Primary atom site location: structure-invariant direct methods
Least-squares matrix: full	Secondary atom site location: difference Fourier map
*R*[*F*^2^ > 2σ(*F*^2^)] = 0.039	Hydrogen site location: inferred from neighbouring sites
*wR*(*F*^2^) = 0.105	H atoms treated by a mixture of independent and constrained refinement
*S* = 1.04	*w* = 1/[σ^2^(*F*_o_^2^) + (0.061*P*)^2^] where *P* = (*F*_o_^2^ + 2*F*_c_^2^)/3
3600 reflections	(Δ/σ)_max_ = 0.001
249 parameters	Δρ_max_ = 0.26 e Å^−^^3^
0 restraints	Δρ_min_ = −0.18 e Å^−^^3^

### Special details

**Table:**

Geometry. Bond distances, angles *etc*. have been calculated using the rounded fractional coordinates. All su's are estimated from the variances of the (full) variance-covariance matrix. The cell e.s.d.'s are taken into account in the estimation of distances, angles and torsion angles
Refinement. Refinement on *F*^2^ for ALL reflections except those flagged by the user for potential systematic errors. Weighted *R*-factors *wR* and all goodnesses of fit *S* are based on *F*^2^, conventional *R*-factors *R* are based on *F*, with *F* set to zero for negative *F*^2^. The observed criterion of *F*^2^ > σ(*F*^2^) is used only for calculating -*R*-factor-obs *etc*. and is not relevant to the choice of reflections for refinement. *R*-factors based on *F*^2^ are statistically about twice as large as those based on *F*, and *R*-factors based on ALL data will be even larger.

### Fractional atomic coordinates and isotropic or equivalent isotropic displacement parameters (Å^2^)

**Table:**

	*x*	*y*	*z*	*U*_iso_*/*U*_eq_	
O1	0.23194 (8)	−0.07143 (8)	0.87203 (6)	0.0383 (3)	
O2	0.49696 (7)	0.03714 (8)	1.12916 (6)	0.0323 (3)	
O3	0.72539 (8)	0.62118 (8)	0.93267 (6)	0.0344 (3)	
N1	0.39904 (9)	0.18280 (9)	0.94790 (7)	0.0266 (3)	
N2	0.50300 (9)	0.22036 (9)	1.01218 (8)	0.0266 (3)	
C1	0.31024 (11)	−0.05076 (11)	1.09863 (9)	0.0270 (4)	
C2	0.35221 (12)	−0.15824 (11)	1.15131 (9)	0.0300 (4)	
C3	0.27939 (13)	−0.22881 (12)	1.18252 (10)	0.0376 (4)	
C4	0.16617 (13)	−0.18947 (13)	1.16436 (10)	0.0410 (5)	
C5	0.12488 (12)	−0.08089 (13)	1.11449 (10)	0.0390 (5)	
C6	0.19590 (11)	−0.01201 (12)	1.08006 (9)	0.0329 (4)	
C7	0.39245 (11)	0.02418 (11)	1.06834 (9)	0.0266 (4)	
C8	0.34628 (10)	0.08883 (11)	0.96964 (9)	0.0261 (3)	
C9	0.24507 (11)	0.03874 (11)	0.88019 (9)	0.0283 (4)	
C10	0.16409 (10)	0.12118 (11)	0.79933 (9)	0.0285 (4)	
C11	0.10610 (11)	0.07481 (12)	0.69998 (9)	0.0341 (4)	
C12	0.02797 (12)	0.14692 (14)	0.62317 (10)	0.0401 (5)	
C13	0.00466 (12)	0.26359 (14)	0.64446 (10)	0.0431 (5)	
C14	0.06158 (12)	0.31001 (13)	0.74235 (10)	0.0401 (5)	
C15	0.14183 (11)	0.23937 (12)	0.81968 (10)	0.0322 (4)	
C16	0.55535 (10)	0.32336 (11)	0.98885 (9)	0.0246 (4)	
C17	0.49391 (10)	0.39449 (10)	0.90350 (9)	0.0260 (4)	
C18	0.54880 (11)	0.49364 (11)	0.88215 (9)	0.0277 (4)	
C19	0.66431 (11)	0.52350 (11)	0.94650 (9)	0.0272 (4)	
C20	0.72508 (11)	0.45238 (11)	1.03236 (9)	0.0292 (4)	
C21	0.67143 (11)	0.35237 (11)	1.05282 (9)	0.0282 (4)	
C22	0.66773 (12)	0.69076 (11)	0.84126 (9)	0.0338 (4)	
H1	0.5436 (12)	0.1775 (13)	1.0677 (11)	0.041 (4)*	
H2	0.43120	−0.18360	1.16610	0.0360*	
H3	0.30730	−0.30380	1.21620	0.0450*	
H4	0.11640	−0.23720	1.18620	0.0490*	
H5	0.04760	−0.05330	1.10370	0.0470*	
H6	0.16650	0.06130	1.04400	0.0390*	
H11	0.12040	−0.00600	0.68540	0.0410*	
H12	−0.00980	0.11610	0.55550	0.0480*	
H13	−0.05060	0.31210	0.59180	0.0520*	
H14	0.04570	0.39040	0.75660	0.0480*	
H15	0.18160	0.27180	0.88660	0.0390*	
H17	0.41430	0.37510	0.85980	0.0310*	
H18	0.50720	0.54150	0.82310	0.0330*	
H20	0.80400	0.47290	1.07710	0.0350*	
H21	0.71390	0.30320	1.11070	0.0340*	
H22A	0.64540	0.63760	0.78130	0.0510*	
H22B	0.72190	0.75360	0.83730	0.0510*	
H22C	0.59640	0.72870	0.84260	0.0510*	

### Atomic displacement parameters (Å^2^)

**Table:**

	*U*^11^	*U*^22^	*U*^33^	*U*^12^	*U*^13^	*U*^23^
O1	0.0453 (6)	0.0278 (5)	0.0359 (5)	−0.0048 (4)	0.0103 (4)	0.0012 (4)
O2	0.0266 (5)	0.0370 (5)	0.0308 (5)	0.0004 (4)	0.0089 (4)	0.0049 (4)
O3	0.0341 (5)	0.0326 (5)	0.0361 (5)	−0.0065 (4)	0.0137 (4)	0.0022 (4)
N1	0.0258 (6)	0.0266 (6)	0.0273 (5)	0.0003 (4)	0.0105 (5)	−0.0019 (4)
N2	0.0252 (6)	0.0283 (6)	0.0248 (5)	−0.0007 (4)	0.0086 (5)	0.0016 (4)
C1	0.0305 (7)	0.0269 (7)	0.0237 (6)	−0.0019 (5)	0.0111 (5)	−0.0031 (5)
C2	0.0370 (7)	0.0303 (7)	0.0242 (6)	−0.0004 (5)	0.0139 (6)	−0.0023 (5)
C3	0.0543 (9)	0.0317 (7)	0.0291 (6)	−0.0062 (6)	0.0191 (6)	−0.0013 (6)
C4	0.0473 (9)	0.0464 (9)	0.0353 (7)	−0.0178 (7)	0.0228 (7)	−0.0053 (6)
C5	0.0316 (8)	0.0520 (9)	0.0355 (7)	−0.0072 (6)	0.0156 (6)	−0.0072 (6)
C6	0.0314 (7)	0.0344 (7)	0.0319 (7)	−0.0002 (6)	0.0117 (6)	−0.0010 (6)
C7	0.0278 (7)	0.0251 (6)	0.0279 (6)	0.0028 (5)	0.0122 (6)	−0.0016 (5)
C8	0.0263 (6)	0.0249 (6)	0.0280 (6)	0.0011 (5)	0.0119 (5)	0.0007 (5)
C9	0.0294 (7)	0.0289 (7)	0.0289 (6)	−0.0019 (5)	0.0142 (5)	0.0018 (5)
C10	0.0223 (6)	0.0343 (7)	0.0285 (6)	−0.0033 (5)	0.0099 (5)	0.0045 (5)
C11	0.0305 (7)	0.0371 (8)	0.0327 (7)	−0.0066 (6)	0.0107 (6)	0.0014 (6)
C12	0.0322 (8)	0.0541 (9)	0.0295 (7)	−0.0070 (6)	0.0076 (6)	0.0046 (6)
C13	0.0303 (8)	0.0575 (10)	0.0386 (8)	0.0069 (7)	0.0107 (6)	0.0189 (7)
C14	0.0362 (8)	0.0423 (8)	0.0449 (8)	0.0098 (6)	0.0194 (7)	0.0104 (6)
C15	0.0296 (7)	0.0372 (8)	0.0317 (7)	0.0017 (6)	0.0143 (6)	0.0037 (6)
C16	0.0261 (7)	0.0251 (7)	0.0251 (6)	0.0007 (5)	0.0128 (5)	−0.0022 (5)
C17	0.0236 (6)	0.0281 (7)	0.0251 (6)	0.0003 (5)	0.0086 (5)	−0.0032 (5)
C18	0.0296 (7)	0.0283 (7)	0.0255 (6)	0.0022 (5)	0.0113 (5)	0.0011 (5)
C19	0.0285 (7)	0.0265 (6)	0.0305 (6)	−0.0015 (5)	0.0158 (5)	−0.0036 (5)
C20	0.0228 (6)	0.0329 (7)	0.0300 (6)	0.0000 (5)	0.0086 (5)	−0.0030 (5)
C21	0.0278 (7)	0.0310 (7)	0.0244 (6)	0.0039 (5)	0.0090 (5)	0.0012 (5)
C22	0.0379 (8)	0.0295 (7)	0.0382 (7)	−0.0017 (6)	0.0197 (6)	0.0027 (6)

### Geometric parameters (Å, °)

**Table:**

O1—C9	1.2280 (15)	C16—C21	1.3884 (19)
O2—C7	1.2368 (16)	C16—C17	1.3890 (17)
O3—C19	1.3743 (16)	C17—C18	1.3839 (18)
O3—C22	1.4311 (15)	C18—C19	1.3866 (19)
N1—N2	1.3062 (15)	C19—C20	1.3922 (17)
N1—C8	1.3259 (16)	C20—C21	1.3776 (18)
N2—C16	1.4120 (16)	C2—H2	0.9500
N2—H1	0.884 (14)	C3—H3	0.9500
C1—C6	1.391 (2)	C4—H4	0.9500
C1—C7	1.4988 (19)	C5—H5	0.9500
C1—C2	1.3893 (17)	C6—H6	0.9500
C2—C3	1.389 (2)	C11—H11	0.9500
C3—C4	1.381 (2)	C12—H12	0.9500
C4—C5	1.383 (2)	C13—H13	0.9500
C5—C6	1.389 (2)	C14—H14	0.9500
C7—C8	1.4725 (17)	C15—H15	0.9500
C8—C9	1.4853 (18)	C17—H17	0.9500
C9—C10	1.4927 (17)	C18—H18	0.9500
C10—C11	1.4012 (17)	C20—H20	0.9500
C10—C15	1.3892 (18)	C21—H21	0.9500
C11—C12	1.3826 (19)	C22—H22A	0.9800
C12—C13	1.381 (2)	C22—H22B	0.9800
C13—C14	1.3833 (19)	C22—H22C	0.9800
C14—C15	1.3872 (19)		
			
C19—O3—C22	116.93 (10)	C18—C19—C20	119.50 (12)
N2—N1—C8	121.26 (10)	C19—C20—C21	120.34 (12)
N1—N2—C16	120.15 (10)	C16—C21—C20	120.04 (11)
N1—N2—H1	119.9 (10)	C1—C2—H2	120.00
C16—N2—H1	119.7 (10)	C3—C2—H2	120.00
C2—C1—C6	119.63 (13)	C2—C3—H3	120.00
C2—C1—C7	118.74 (13)	C4—C3—H3	120.00
C6—C1—C7	121.55 (11)	C3—C4—H4	120.00
C1—C2—C3	120.37 (14)	C5—C4—H4	120.00
C2—C3—C4	119.68 (13)	C4—C5—H5	120.00
C3—C4—C5	120.28 (14)	C6—C5—H5	120.00
C4—C5—C6	120.25 (14)	C1—C6—H6	120.00
C1—C6—C5	119.74 (12)	C5—C6—H6	120.00
O2—C7—C8	120.42 (12)	C10—C11—H11	120.00
O2—C7—C1	119.64 (11)	C12—C11—H11	120.00
C1—C7—C8	119.80 (11)	C11—C12—H12	120.00
N1—C8—C9	114.35 (10)	C13—C12—H12	120.00
N1—C8—C7	124.47 (11)	C12—C13—H13	120.00
C7—C8—C9	120.47 (11)	C14—C13—H13	120.00
O1—C9—C10	120.72 (11)	C13—C14—H14	120.00
C8—C9—C10	120.27 (11)	C15—C14—H14	120.00
O1—C9—C8	118.95 (11)	C10—C15—H15	120.00
C11—C10—C15	119.45 (12)	C14—C15—H15	120.00
C9—C10—C11	117.85 (11)	C16—C17—H17	120.00
C9—C10—C15	122.66 (11)	C18—C17—H17	120.00
C10—C11—C12	119.89 (12)	C17—C18—H18	120.00
C11—C12—C13	120.29 (12)	C19—C18—H18	120.00
C12—C13—C14	120.13 (13)	C19—C20—H20	120.00
C13—C14—C15	120.15 (13)	C21—C20—H20	120.00
C10—C15—C14	120.06 (12)	C16—C21—H21	120.00
C17—C16—C21	119.89 (11)	C20—C21—H21	120.00
N2—C16—C17	121.54 (11)	O3—C22—H22A	109.00
N2—C16—C21	118.56 (11)	O3—C22—H22B	109.00
C16—C17—C18	119.91 (12)	O3—C22—H22C	109.00
C17—C18—C19	120.30 (11)	H22A—C22—H22B	110.00
O3—C19—C20	115.97 (11)	H22A—C22—H22C	109.00
O3—C19—C18	124.52 (11)	H22B—C22—H22C	109.00
			
C22—O3—C19—C18	−4.76 (18)	C7—C8—C9—O1	−30.36 (19)
C22—O3—C19—C20	175.85 (11)	C7—C8—C9—C10	152.35 (12)
C8—N1—N2—C16	−177.67 (11)	O1—C9—C10—C11	−24.5 (2)
N2—N1—C8—C7	4.91 (19)	O1—C9—C10—C15	153.45 (14)
N2—N1—C8—C9	−165.34 (11)	C8—C9—C10—C11	152.71 (13)
N1—N2—C16—C17	6.82 (18)	C8—C9—C10—C15	−29.3 (2)
N1—N2—C16—C21	−172.50 (12)	C9—C10—C11—C12	178.39 (13)
C6—C1—C2—C3	2.05 (18)	C15—C10—C11—C12	0.3 (2)
C7—C1—C2—C3	178.65 (11)	C9—C10—C15—C14	−177.10 (13)
C2—C1—C6—C5	0.13 (18)	C11—C10—C15—C14	0.9 (2)
C7—C1—C6—C5	−176.37 (11)	C10—C11—C12—C13	−1.5 (2)
C2—C1—C7—O2	−41.22 (17)	C11—C12—C13—C14	1.6 (2)
C2—C1—C7—C8	143.16 (12)	C12—C13—C14—C15	−0.4 (2)
C6—C1—C7—O2	135.31 (13)	C13—C14—C15—C10	−0.9 (2)
C6—C1—C7—C8	−40.31 (17)	N2—C16—C17—C18	−178.94 (12)
C1—C2—C3—C4	−2.43 (19)	C21—C16—C17—C18	0.37 (19)
C2—C3—C4—C5	0.6 (2)	N2—C16—C21—C20	−179.88 (12)
C3—C4—C5—C6	1.6 (2)	C17—C16—C21—C20	0.79 (19)
C4—C5—C6—C1	−1.95 (19)	C16—C17—C18—C19	−1.03 (19)
O2—C7—C8—N1	−16.2 (2)	C17—C18—C19—O3	−178.83 (12)
O2—C7—C8—C9	153.45 (12)	C17—C18—C19—C20	0.53 (19)
C1—C7—C8—N1	159.35 (12)	O3—C19—C20—C21	−179.95 (12)
C1—C7—C8—C9	−30.96 (18)	C18—C19—C20—C21	0.6 (2)
N1—C8—C9—O1	140.32 (13)	C19—C20—C21—C16	−1.3 (2)
N1—C8—C9—C10	−36.97 (18)		

### Hydrogen-bond geometry (Å, °)

**Table:**

*D*—H···*A*	*D*—H	H···*A*	*D*···*A*	*D*—H···*A*
N2—H1···O2	0.884 (14)	1.977 (15)	2.6427 (14)	131.0 (14)
C15—H15···O3^i^	0.95	2.64	3.5859 (16)	174
C21—H21···O1^ii^	0.95	2.63	3.3441 (15)	132

Symmetry codes: (i) −*x*+1, −*y*+1, −*z*+2; (ii) −*x*+1, −*y*, −*z*+2.
